# Supplementary material for: In Vitro Influence of a Chemically Characterized Hippophae rhamnoides L. Fruit Extract on Healthy and Constipated Human Gut Microbiota Functionality and Aquaporin-3 Expression
Source: Foods. 2025 Nov 6;14(21):3800. doi: 10.3390/foods14213800 (PMC12607761; doi:10.3390/foods14213800)
Supplement: Supplementary file 1 [file foods-14-03800-s001.zip › foods-3921155-supplementary.pptx]

## Slide 1
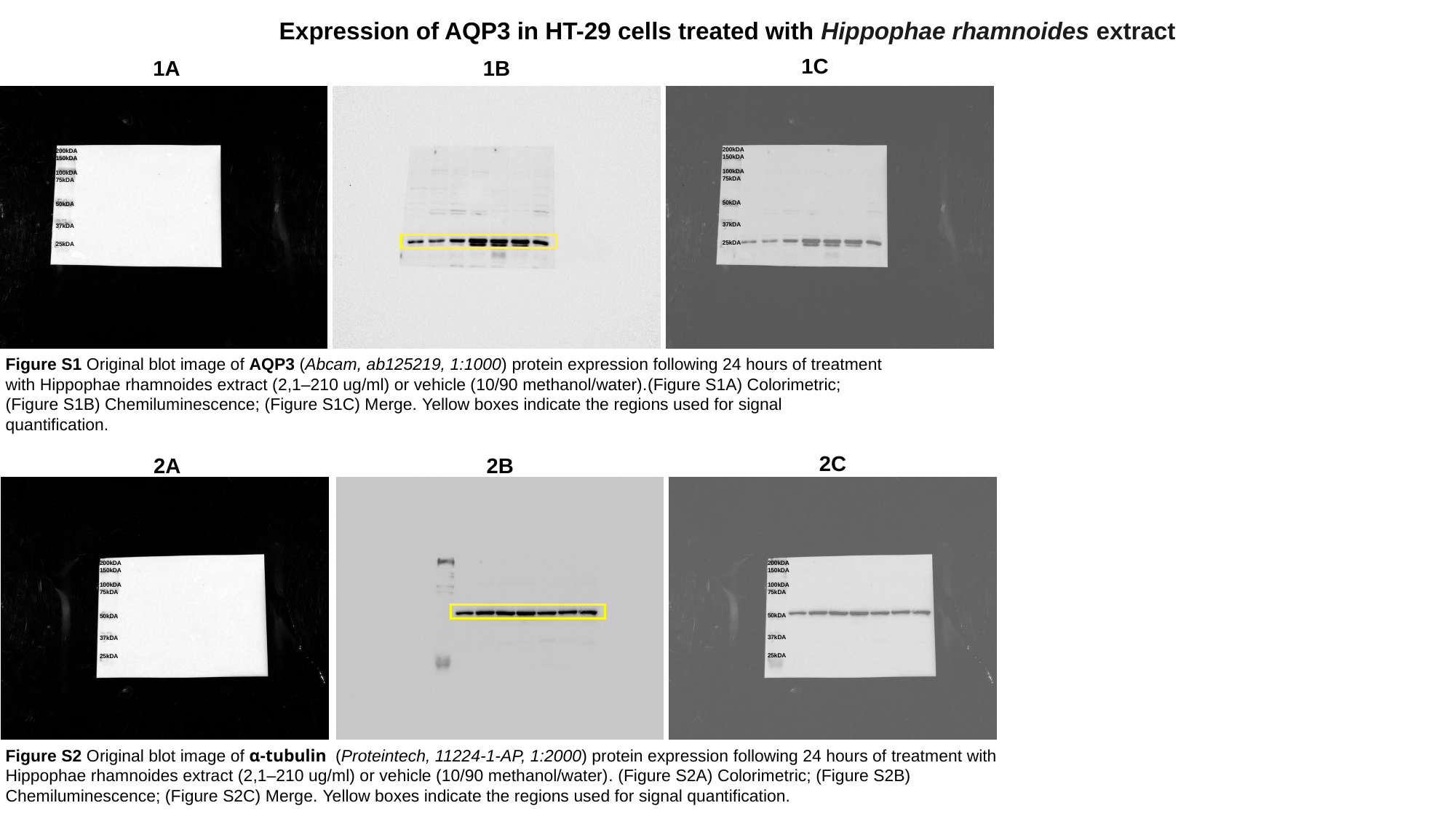

Expression of AQP3 in HT-29 cells treated with Hippophae rhamnoides extract
1C
1A
1B
200kDA
150kDA
100kDA
75kDA
50kDA
37kDA
25kDA
200kDA
150kDA
100kDA
75kDA
50kDA
37kDA
25kDA
Figure S1 Original blot image of AQP3 (Abcam, ab125219, 1:1000) protein expression following 24 hours of treatment with Hippophae rhamnoides extract (2,1–210 ug/ml) or vehicle (10/90 methanol/water).(Figure S1A) Colorimetric; (Figure S1B) Chemiluminescence; (Figure S1C) Merge. Yellow boxes indicate the regions used for signal quantification.
2C
2A
2B
200kDA
150kDA
100kDA
75kDA
50kDA
37kDA
25kDA
200kDA
150kDA
100kDA
75kDA
50kDA
37kDA
25kDA
Figure S2 Original blot image of α-tubulin (Proteintech, 11224-1-AP, 1:2000) protein expression following 24 hours of treatment with Hippophae rhamnoides extract (2,1–210 ug/ml) or vehicle (10/90 methanol/water). (Figure S2A) Colorimetric; (Figure S2B) Chemiluminescence; (Figure S2C) Merge. Yellow boxes indicate the regions used for signal quantification.
